# Electrospinning Proteins for Wound Healing Purposes: Opportunities and Challenges

**DOI:** 10.3390/pharmaceutics13010004

**Published:** 2020-12-22

**Authors:** Alma Akhmetova, Andrea Heinz

**Affiliations:** LEO Foundation Center for Cutaneous Drug Delivery, Department of Pharmacy, University of Copenhagen, 2100 Copenhagen, Denmark; alma.akhmetova@sund.ku.dk

**Keywords:** antibacterial, antimicrobial, biomaterial, infection, microfibers, nanofibers, scaffold, tissue engineering, wound dressing

## Abstract

With the growth of the aging population worldwide, chronic wounds represent an increasing burden to healthcare systems. Wound healing is complex and not only affected by the patient’s physiological conditions, but also by bacterial infections and inflammation, which delay wound closure and re-epithelialization. In recent years, there has been a growing interest for electrospun polymeric wound dressings with fiber diameters in the nano- and micrometer range. Such wound dressings display a number of properties, which support and accelerate wound healing. For instance, they provide physical and mechanical protection, exhibit a high surface area, allow gas exchange, are cytocompatible and biodegradable, resemble the structure of the native extracellular matrix, and deliver antibacterial agents locally into the wound. This review paper gives an overview on cytocompatible and biodegradable fibrous wound dressings obtained by electrospinning proteins and peptides of animal and plant origin in recent years. Focus is placed on the requirements for the fabrication of such drug delivery systems by electrospinning as well as their wound healing properties and therapeutic potential. Moreover, the incorporation of antimicrobial agents into the fibers or their attachment onto the fiber surface as well as their antimicrobial activity are discussed.

## 1. Wound Healing and Electrospun Wound Dressings

The wound healing process is associated with four overlapping and well-orchestrated stages: homeostasis, inflammation, proliferation and remodeling. Each stage involves a cascade of events to ensure prevention of blood loss, elimination of bacterial contamination, regeneration and formation of a new skin tissue, respectively. A variation from the norm in this process results in a delay or prolongation of any of the healing stages, which in turn leads to impaired healing [1]. The interruption in the healing process may occur for a number of reasons connected to one’s lifestyle and health condition. For example, smoking, malnutrition, obesity, low mobility, neuropathy, diabetes, vascular diseases and skin disorders have been linked to the increasing chronicity of wounds, where healing has not been achieved within 3–6 weeks [2,3,4].

**Figure 1:**
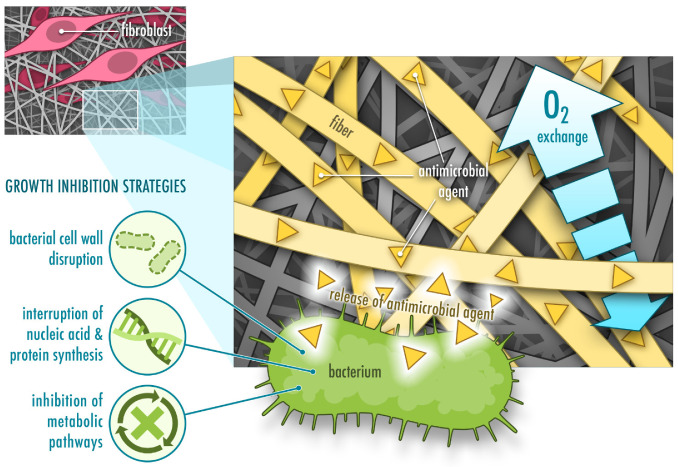
Functions of protein-based nanofibrous mats with incorporated antimicrobial agents for wound healing. They allow fibroblast adhesion (often through cell-recognizing motifs the fiber carries), oxygen exchange and show bacteriostatic or bactericidal activity.

Compromised wound healing represents a complex problem of multiple dependent molecular and cellular processes that are closely intertwined. A slight dysregulation in those processes leads to a development of a chronic non-healing condition, which requires a combinational approach of diverse strategies to facilitate healing. Different polymeric wound dressings have been created to supply favorable environment for wound healing, to absorb exudate, allow vapor exchange across the scaffold, maintain moist conditions, provide mechanical support and protect from further bacterial contamination. Such wound dressings have also been employed to deliver active agents such as antibiotics, antiseptics, anti-inflammatory drugs and biomolecules to direct the healing process to reach complete healing [5,6] (Figure 1).

The leading causes of non-healing chronic wounds are infection, pathological inflammation and formation of biofilms [2,6]. Therefore, wound care usually comprises of debridement followed by antimicrobial treatment and application of wound dressings. Debridement is required to clean the wound bed from exudate, necrotic tissue and bacterial load. Antimicrobial treatment prevents further bacterial growth and formation of biofilms. Antimicrobial agents usually follow one or several strategies to attack bacterial cells, including disruption of the bacterial cell wall, interruption of nucleic acid and protein synthesis, and dysregulation of metabolic pathways [7] (Figure 1). Antiseptics, antibiotics or other biomolecules are either applied directly or incorporated into a wound dressing [6]. In comparison to systemic administration of antimicrobial treatments, topical application requires lower concentrations, displays fewer side effects and lowers the risk of developing antibiotic resistance [6,8]. Topical application of antimicrobial agents such as antibiotics often combines a rapid initial release to kill bacteria or inhibit bacterial growth followed by a slower release to prevent further bacterial growth [9]. In order to prevent development of microbial resistance to antibiotics, silver nanoparticles have been used in certain materials for wound healing instead of antibiotics. However, recent studies demonstrate that bacterial resistance also occurs against silver nanoparticles due to an induction of nanoparticle aggregation as a result of the production of adhesive proteins by the bacteria. This problem can be overcome by additional stabilization of the nanoparticles by surfactants or polymers [10,11].

A variety of wound dressings facilitating wound healing are currently available on the market and new advanced materials are being developed (e.g., films, hydrogels, foams, hydrocolloids and nanoparticles). In particular, large research efforts have been directed to fabricate nanofibers [5,12,13]. Unlike other types of biomaterials, nanofibers stand out due to their unique structure and the tunability of their physical and mechanical properties. Their versatility and the easy fabrication process facilitate obtaining materials with desired characteristics for the complex wound healing process. High surface area and homogenous drug distribution makes nanofibers attractive as drug delivery systems with high drug loading capacity and controlled release. Resemblance of nanofibers to collagen or elastin fibers in the extracellular matrix (ECM) of healthy skin allows them to provide additional support for fibroblasts and keratinocytes, which adhere to the fibers, migrate across the wound bed and help regenerate and close the damaged tissue. Modifications of the surface morphology of nanofibers and the porosity of the nanofibrous matrix further promote adherence and migration of these cells [12] (Figure 1). However, even though electrospun fibers are often have a high porosity, this property is dependent on the fiber diameter and is difficult to control. This may also limit cell penetration into the scaffold in some cases [14].

**Figure 2:**
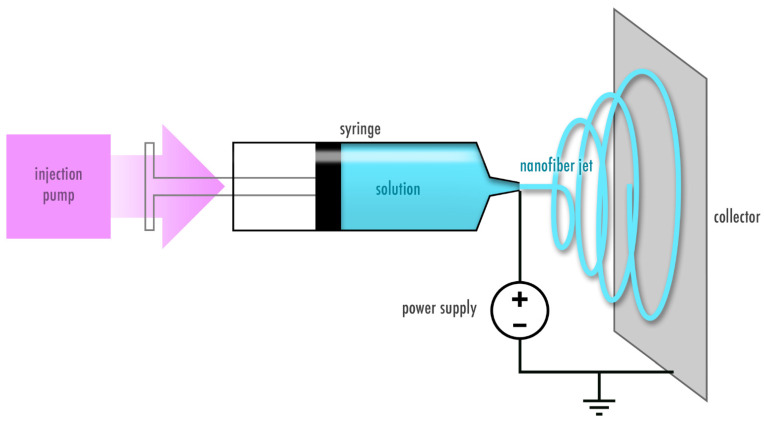
Electrospinning process. A polymer solution is subjected to a high voltage output to create a polymer jet that deposits as dry fibers on the collector.

A variety of methods to fabricate fibers have been developed over the years and mainly include solution and melt electrospinning [15]. This review focuses on nanofibers created from protein solutions using the solution electrospinning process. Electrospinning is based on applying a high voltage to a polymer solution to transform a drop at the needle tip into a cone shape in order to generate a jet. The ejected jet undergoes a number of instabilities, during which the solvent from the solution is evaporated and dry fibers are collected on the grounded or oppositely charged plate. The process is shown in Figure 2. The morphology, diameter size and distribution of electrospun fibers can be adjusted and tuned according to the solution (e.g., concentration, molecular weight, viscosity, conductivity, surface tension, dielectric constant, evaporation rate and dipole moment) and process parameters (e.g., temperature, humidity, flow rate, voltage and working distance) [16]. For example, larger fiber diameter is often associated with higher flow rate, higher applied voltage and lower distance between the needle tip and the collector. However, there are exceptions to these rules as for instance a higher voltage may lead to more solution deposition [15]. Therefore both, the properties of the solution and the process parameters should be considered during optimization of the electrospinning process [16].

**Figure 3:**
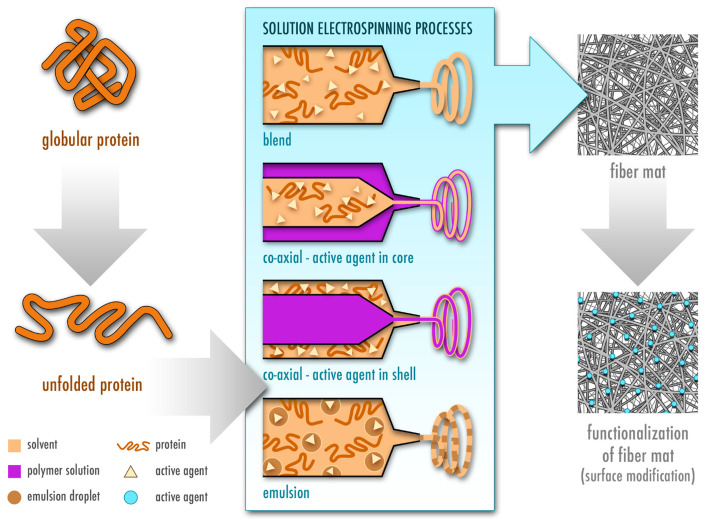
Fabrication of protein-based electrospun fiber mats by different types of solution electrospinning, namely blend, co-axial and emulsion electrospinning. The protein is first dissolved in a volatile solvent and starts unfolding, which is a prerequisite for successful electrospinning of proteins. In blend electrospinning, the active agent is directly added to the polymeric protein solution. In co-axial electrospinning, the active agent is either dissolved in the shell or the core solution. In addition to the protein solution, a second natural or synthetic polymer is used in co-axial electrospinning. In emulsion electrospinning, the drug is dissolved in the emulsion droplets (inner phase). In addition, the fibrous mat can be functionalized by adding the active agent after electrospinning.

The most widely used type of solution electrospinning is single-nozzle electrospinning (also known as blend electrospinning), which itself has a few subcategories with some variations including co-axial and emulsion electrospinning [15]. These techniques are commonly employed to incorporate drugs, including active biomolecules, and are summarized in Figure 3. In blend electrospinning, the drug is mixed into the polymer solution—in this case the protein solution—directly. In contrast, in co-axial electrospinning two different solutions are used, and the drug is incorporated either in the outer (shell) or inner (core) solution [17]. Additionally, the drug can be incorporated into an emulsion to be electrospun, where the final product is similar to that obtained by co-axial electrospinning due to the lengthening of the emulsion within the jet, which creates a core-shell structure [18,19]. The electrospinning technique is chosen depending on the solubility of the polymer in a particular solvent, as well as its stability during the electrospinning process and the desired release kinetics of the electrospun nanofibers. During blend electrospinning, organic and sometimes highly toxic solvents are commonly used and may affect structure, stability and activity of the drug. Therefore, co-axial and emulsion electrospinning provide an alternative, where the drug can be dissolved in a more favorable solvent [20,21,22,23]. Nevertheless, all of these techniques involve high voltage, which may potentially damage the therapeutic agent [22,23]. In such a case, there is another method that is based on a functionalization of the nanofiber surface after electrospinning by attachment of the drug (Figure 3). However, a drawback of functionalization of the fibers as compared to other methods, where the drug is incorporated into the fibers, is that the drug lacks a coating material, which normally acts as a protective layer to provide longer shelf life [21].

## 2. Proteins as a Promising Starting Material for Electrospun Wound Dressings

The initial use of synthetic polymers in electrospinning has noticeably shifted towards implementation of natural polymers such as proteins and carbohydrates [24]. In comparison to synthetic polymers such as polylactic acid (PLA) [25] and polyurethane (PU) [26,27], natural polymers do not purely rely on the use of harsh and toxic organic solvents for dissolution. Therefore, they provide an environmentally friendly alternative, which may additionally offer better drug stability and activity as compared to pharmaceutical standard formulations, safer manufacturing and the possibility of an application on skin [28]. However, this comes at a cost of easy fabrication and reproducibility. Evaporation rate, surface tension and conductivity of the employed solvent greatly affect electrospinnability of the protein solution [15]. Moreover, electrospinning of proteins is more challenging due to the intrinsic variations in complexity of their structures, molecular weight, surface charge as well as ionic, hydrogen and disulfide bonds [29,30]. The electrospinnability of proteins depends not only on their solubility in a specific solvent, but also on the degree of protein unfolding in a particular solvent [29,31] and chain entanglement [31,32] (Figure 3). The choice of the solvent further affects crystallinity, mechanical properties, fiber size and morphology [29,31]. Therefore, the addition of synthetic polymers is often necessary to electrospin the solution continuously and without artifacts [24,33].

Proteins demonstrate attractive features as antimicrobial delivery system due to their natural origin, fast biodegradability and cytocompatibility [24,34]. Proteins used in electrospinning for wound healing applications are mainly obtained from two distinct sources: plants and animals [13,33]. Their stability, activity and degradation depend on the protein size, chemical structure, isolation and purification processes [5,35]. Different methods for protein extraction and purification may affect the obtained raw material’s purity and composition [5,36,37], which in turn impacts reproducibility of the electrospinning process and properties of the final product [38].

Some of the main differences between plant- and animal-based proteins are their availability and price. Plant proteins tend to be available in larger amounts and at a lower cost [31,34,39,40]. As compared to synthetic polymers, proteins are in general more challenging to electrospin due to their heterogeneity in structure and surface charge, solvent-dependent protein unfolding and low viscosity, which lead to a non-continuous electrospinning process and formation of beads [24,29]. Moreover, the final product may lack stability in water, resulting in a loss of fiber structure [41,42]. To compensate for these drawbacks, different strategies have been implemented that include the use of cross-linking agents, toxic organic solvents and addition of synthetic polymers [33].

## 3. Electrospinning of Plant-Derived Proteins for Wound Healing Purposes

Plant proteins that have been used to prepare electrospun wound dressings alone or in combination with other natural and/or synthetic polymers are summarized in Table 1. These include zein protein, soy protein and pea protein.

### 3.1. Zein Protein

Among the available plant-derived proteins, zein protein is extensively being used for a variety of drug delivery systems such as films, gels, nanoparticles and nanofibers [56,57]. Its self-assembling nature and insolubility in water have made it interesting for application in surface protection for food packaging, a variety of sensors and air filtration [58] as well as vaccines and tissue engineering. Zein protein is extracted from corn seed and is categorized into prolamines (α and δ) and glutelins (β and γ). It does not carry a nutritional value due to the lack of the key amino acids lysine and tryptophan required for human diet. However, the high amount of glutamic acid, proline, alanine and leucine in zein protein are responsible for its hydrophobic nature [57]. Zein may carry genes that cause immunogenic reaction [59], and studies on oral [60] and intramuscular administration [61] reveal controversial results. Despite its wide use, the tertiary structure of zein still remains unknown and is not available on the Protein Databank. Only a few studies have attempted to hypothesize on its structure, providing varying results from antiparallel helices forming a cylinder [62,63], triple superhelices [64] to antiparallel helices arranged in hexagonal repeats [65].

For wound healing applications, zein protein has mostly been used either as film [66,67] or nanofibrous scaffold [26,50]. One of the major advantages of electrospinning zein is that the use of toxic solvents and cross-linkers can be avoided due to its sufficient solubility in aqueous ethanol and self-assembling nature, respectively [56,68,69]. However, aqueous ethanol is not an ideal solvent for electrospinning zein protein [70]. Due to its high evaporation rate, it leads to needle clogging, formation of ribbon-shaped fibers and results in poor water stability of the fibers, which in turn leads to the loss of porous structure of the fiber mat upon contact with water [41,42]. This can be overcome either by cross-linking zein with UV light [71] or by co-axial electrospinning with ethanol [41] or polyethylene oxide (PEO) as a shell solution [54]. The behavior of zein in aqueous ethanol solution is described in more detail elsewhere [72]. There are various studies, which focus on electrospinning zein to produce antimicrobial wound dressings (Table 1). Zein has been electrospun alone [45,50,51,52,53], together with synthetic polymers such as polycaprolactone (PCL) [47,48], PEO [54], PU [27] and PLA [55] as well as with natural polymers and substances including gum arabic [47,48], hyaluronic acid [46], cinnamaldehyde [26] and gum traganth [55]. Antimicrobial agents that have been incorporated into zein-based fibers include a wide range of antibiotics [26,45,46,49,51,54,55], antibacterial nanoparticles [27,50,52] as well as antimicrobial plant extracts [46,73].

It is worth mentioning that peptides produced from thermolysin-based hydrolysis of zein protein are able to induce the production of angiotensin converting enzyme inhibitor (ACEI) [74]. Inhibition of angiotensin receptors or conversion of angiotensin I to angiotensin II by ACEI have shown to accelerate wound closure and prevent scar tissue formation when ACEIs are administered topically [75] and orally [73], respectively. Further, more structurally organized collagen fibers resembling normal skin structure were observed at the wound site due to the inhibition of tumor growth factor-β1 expression, fibroblast proliferation and collagen production after ACEI administration [73,75].

### 3.2. Soy Protein

Unlike zein, soy proteins are widely researched not only for food packaging [76], but also for their use in food products [77,78] due to their high availability and affordability [31]. However, in comparison to zein, soy protein has not been reported to be electrospun on its own. Polymers such as polyvinyl alcohol (PVA) [30,79], PCL [80] and PEO [44,76,81,82] are required to achieve a bead-free morphology and overcome brittleness of the material (Table 1). Only a limited number of studies have, therefore, focused on applying soy-based nanofibers for wound healing [44,81,82,83], even though soy protein contains reactive amino acid residues such as arginine, glycine, aspartic acid and glutamine that facilitate wound healing through cell attachment and proliferation [83,84,85,86]. Higher cell proliferation in vitro has been demonstrated in electrospun soy protein in comparison to a solvent cast film, which has been attributed to the porous nanofibrous matrix that allows better nutrient access [83].

Soy protein isolates reach approximately 90% purity, and the presence of plant estrogens and isoflavones in the isolates demonstrates controversial biological impact [87,88]. On the one hand, isoflavones possess valuable antioxidant, antimicrobial and anti-inflammatory properties [88]. In fact, the innate antimicrobial effect of soy protein against *E. coli* and *S. aureus* has been shown (Table 1) [44]; however, nobody has attempted to incorporate antimicrobial agents into soy protein-based nanofibers yet. On the other hand, isoflavones have been shown to have carcinogenic and immunosuppressive effects. The latter negatively affect the production of nitric oxide [88], leading to delayed wound healing [89]. Moreover, soy protein isolates have been shown to contain two major allergens, glycinin and β-conglycinin [38,90]. However, to the best of our knowledge no study has focused on these side effects associated with the development of soy protein-based wound dressings. Therefore, more studies are required to better understand the influence of soy protein biomaterials on wound healing.

### 3.3. Pea Protein

In comparison to other proteins, pea seed-derived proteins have only recently been electrospun into nanofibers [29,43]. Pea seeds, unlike soybean, contain fewer proteinase inhibitors and less phytic acid, resulting in lower allergic reaction in humans. Pea proteins mostly consist of globulins up to 65% and albumin of around 25% [91]. Electrospinning of pea protein remains a challenge due to the protein’s globular nature and absence of molecular entanglement [43,92]. Despite the addition of PVA [43] or the use of a variety of solvents [29], the fabricated fibers have been demonstrated to show heterogeneous size distribution and the presence of artifacts. There is only one single study that has explored incorporation of an antimicrobial agent (cinnamaldehyde) into pea protein-based nanofibers, which showed a pronounced effect against *E. coli* and *Listeria monocytogenes* (Table 1) [43]. However, even though cinnamaldehyde carries anti-inflammatory, antimicrobial, antifungal and anti-biofilm features, it is a well-known allergen that could cause burning sensation [93,94]. Therefore, its application in wound healing should be considered with caution.

Recent research using artificial intelligence and a deep learning approach predicted a peptide within the pea protein genome with anti-aging properties that has a potential to be used in wound healing. In particular, this peptide has been shown to facilitate proliferation of keratinocytes and induce production of elastin and collagen from fibroblasts. The in vitro wound scratch assay demonstrated promising results of 40% decrease in wound area in comparison to a control after 48 h of incubation [95].

## 4. Electrospinning Animal-Derived Proteins for Wound Healing Purposes

A lot of animal-derived proteins used in electrospinning are obtained from milk, including casein, whey, lactoferrin and lysozyme, or connective tissue, such as collagen and elastin. In comparison to plant-derived proteins that mostly require an incorporation of antimicrobial agents, proteins obtained from milk possess innate antimicrobial properties due to their iron-binding properties [96,97] and ability to disrupt bacterial cell walls [96,98]. Therefore, such proteins carry a dual function as a material basis with antimicrobial effect.

### 4.1. Casein

Casein has recently gained increasing attention as a biodegradable, cytocompatible, self-assembling and highly available starting material for electrospinning. It is derived from cow’s milk, comprises around 80% of the milk’s protein content and is potentially allergenic. Approximately 55% of casein consists of polar amino acid groups, which allow the formation of hydrogen bonds. This property facilitates the formation of films, but also leads to casein’s poor electrospinnability, which is further compromised due to the low viscoelasticity of it in solution [99,100,101,102]. Therefore, electrospinning fibers from casein is only possible upon addition of synthetic polymers such as PEO [99] and PVA [103]. Moreover, the high hydrophilicity of this protein leads to weak mechanical strength and water stability, which requires the use of toxic cross-linking agents such as glutaraldehyde and silane [99,103]. Antimicrobial activity has not been reported for casein, however, oligopeptides released during enzymatic digestion of casein demonstrated inhibition of several bacterial strains [104]. No antimicrobial drugs have been reported to be incorporated into casein-based nanofibers. However, casein-PEO nanofibers with and without silver nanoparticles showed pronounced inhibition zones against *E. coli* and *S. aureus* (Table 2) [105,106].

### 4.2. Whey

Whey is a globular protein found in milk and is obtained as a by-product of processing dairy products such as casein and cheese. Whey proteins mostly consist of β-lactoglobulin and α-lactoglobulin [36], and minor fractions include lactoferrin and lysozyme among others. The source and manufacturing process greatly affect the composition and functionality of whey proteins, which can reach approximately 80% purity. Whey sub-fractions such as lactoferrin pose manufacturing and purification challenges [146]. Whey proteins have a high nutritional value and demonstrate antimicrobial, antioxidant, and anticancer properties [36,147]. Additionally, whey proteins are often used for their foaming, gelling and emulsifying properties [36]. Whey proteins have been electrospun with PEO [148] and PCL [149], however, an incorporation of antimicrobial agents has not been reported yet.

#### 4.2.1. Lactoglobulins

β-Lactoglobulin is a self-assembling protein [150], which potentially bears antimicrobial properties due to its iron-binding ability. It has been electrospun into nanofibers using PEO, but antimicrobial properties have not been investigated [151]. α-Lactoglobulin is also a self-assembling protein [152] and has been electrospun after addition of PEO. α-Lactoglobulin fibers alone did not demonstrate antimicrobial properties, but an incorporation of ampicillin showed time- and concentration-dependent inhibition of *E. coli* (Table 2) [107].

#### 4.2.2. Lactoferrin

Lactoferrin is a globular glycoprotein with nutritional, antimicrobial, anti-inflammatory and anti-oxidant properties [146]. Due to its iron-chelating property, it exerts a wide range of bacteriostatic effects on a variety of pathogens [96,97], including Gram-positive and Gram-negative bacteria, viruses and fungi [153,154]. Lactoferrin is also a part of pre-immune defense system and can be found in different bodily fluids. Apart from its bacteriostatic properties, lactoferrin also has antimicrobial and antibiofilm function due to its ability to directly bind to bacterial membranes and initiate disruption [154]. More recent research shows that lactoferrin also possesses antioxidant and anticancer activities, and is involved in modulation of metabolic system [155]. Similar to other proteins, lactoferrin has been electrospun into fibers after addition of other polymers such as PCL [156], poly(lactic) acid (PLA) [153] and gelatin [108] (Table 2). A synthetic analog of lactoferrin is available as the peptide GRRRRSVQWCA, known as hLF1-11, which possesses similar bactericidal properties [157]. Nevertheless, there is no record of the peptide being used in electrospinning yet. It has, however, already been functionalized on the titanium surface demonstrating inhibition of bacterial growth and reduction in biofilm formation [158].

#### 4.2.3. Lysozyme

Lysozyme is a 14 kDa enzyme with antimicrobial properties due to its ability to hydrolyze polysaccharides in bacterial cell wall [96,98]. It is abundantly present in the liver and bodily fluids such as saliva, tears and milk. Lysozyme is well known for its antimicrobial activity and is widely researched and used in the food industry and biomedical field. It can be obtained from both plants or animals [98], with lysozyme from hen egg white mainly being used in electrospinning. Purification of lysozyme from eggs needs to be carried out carefully because of impurities from other egg proteins that may contaminate the final product and lead to allergic reactions [37]. Lysozyme has either been incorporated into fibers by blend [159,160], co-axial [23] and emulsion electrospinning [161] or has been functionalized on the surface of the fibers [109,162] (Table 2). Lysozyme is not electrospinnable on its own and has been electrospun with a variety of polymers such as PLGA [23] and poly(vinylpyrrolidone) (PVP) [160]. The disruption of molecular conformation and subsequent decrease in activity of lysozyme has been shown during the use of organic solvents such as chloroform and dimethyl formamide and application of high voltage during electrospinning. When lysozyme was separated from organic solvents, and dissolved in aqueous solutions in the case of co-axial and emulsion electrospinning, its activity was retained approximately above 80%, despite the applied electric field [23].

### 4.3. Keratin

Keratin proteins are found in epithelial tissues and can be easily extracted from hair, wool and feathers [110]. They have an ability to self-assemble into fibrous structures [163] and as compared to other epithelial components such as collagen and elastin, keratin is more cytocompatible and non-immunogenic [164,165]. Keratin proteins are insoluble in water, biodegradable, possess strong mechanical properties and contain cell-binding motifs such as the arginine-glycine-aspartic acid (RGD) motif [33,166,167]. These advantages have led to its use in the fabrication of materials for biomedical purposes. Electrospinning keratin is challenging due to its high molecular weight and low viscoelasticity [33]. Therefore, keratin has been electrospun into fibers after addition of other polymers such as PVA [110], PEO [110,168] and chitosan (CS) [111]. Better cytocompatibility is achieved at higher keratin concentrations in the fibers [169]; however, higher keratin concentrations make it more challenging to electrospin and often result in beaded morphology [168,169]. As keratin does not possess an intrinsic antimicrobial property, a few studies have incorporated silver nanoparticles [110], antibiotics such as mupirocin [111] and the bactericidal agent irgasan [168] into keratin-based fibers (Table 2).

### 4.4. Silk Fibroin and Sericin

Silk is a natural biopolymer and product of the secretion process of a number of arthropod lineages, including wasps (nests), silkworms (cocoons) and spiders (webs). It consists of two major components: fibroin and sericin. Silk sericin (SS) is the sticky protein on the outside of silk strands, makes up 15–35% of silk cocoons of the *Bombyx mori* silkworm and is normally removed during extraction of the more versatile silk fibroin (SF) [170]. However, SS exhibits a number of useful properties such as cytocompatibility, biodegradability, moisture absorption as well as antioxidant, antibacterial and UV resistance properties [171]. SS has further been shown to accelerate wound healing and collagen production [172].

SF is the main component of the natural silkworm thread obtained from the *Bombyx mori* silkworm. It contains polypeptide chains with molecular weights between 200 kDa and 350 kDa, which are composed of repetitive blocks of hydrophobic heavy chains and hydrophilic light chains linked by disulfide bonds. In addition, a glycoprotein P25 is non-covalently linked to the heavy and light chains and is responsible for integrity of the structure. Heavy SF forms anti-parallel β-sheets and highly organized crystalline domains due to hydrogen bonding, van der Waals forces and hydrophobic interactions in repetitive domains of the protein. These crystalline domains are cross-linked to an amorphous matrix formed in the non-repetitive domains of the protein, which comprises random coils, β-turns and α-helices. Altogether, this generates a semi-crystalline fishnet structure, in which the crystalline areas absorb pressure and distribute it throughout the entire fibroin network. SF has been increasingly used as a biomaterial for tissue engineering in the last decade due to its availability, low cost, cytocompatibility, bioactivity, biodegradability, thermostability, ideal mechanical properties and low immunogenicity [33,34]. Its low weight (1.3 g cm^−3^) and high tensile strength (~4.8 GPa) make it ideal for the production of electrospun fibers and its oxygen and water vapor permeability allow its use for wound healing purposes. The mechanical properties of SF fibers can also be altered through methanol treatment after electrospinning, which increases β–sheet crystallinity and reduces water solubility. The degradation of SF occurs via enzymatic surface erosion [170]. It is worth noting that SFs from other, so-called non-mulberry silkworms *Antheraea assama* and *Philosamia ricini* possess RGD cell-binding motifs that allow for interactions between the cells and the biomaterial.

To date, SS has only been used in a few electrospinning studies for wound healing purposes and has been blended with other synthetic polymers including PVA [125] and poly(L-lactic acid) (PLLA) [124] and natural polymers such as CS [123,125] to improve the antimicrobial and mechanical properties of the fiber mats (Table 2). It is worth noting that SS-CS nanofibers without addition of an active pharmaceutical component showed antimicrobial activity against both *E. coli* and *B. subtilis* with 100% growth inhibition at a nanofiber concentration of 0.4 mg mL^−1^. Due to the positive surface electrical charge of the composite nanofibers, the antimicrobial activity of the fibers was better against Gram-negative *E. coli* [123]. In contrast to SS, there are multiple studies that have used SF as a starting material for electrospun wound dressings. SF has been electrospun alone [127,130,140,142,143], in combination with synthetic polymers such as PCL [126,134,137], polyethylenimine (PEI) [129], PVA [131,132], poly(L-lactic acid-co-e-caprolactone) [136], PEO [135,137,138,144,145], and in combination with natural polymers such as gelatin [133] and CS as well as its derivatives [128,138,141] to tune the mechanical and physicochemical properties of the fiber mats (Table 2). In many cases, the electrospun SF-based fibers have been post-treated with ethanol, methanol, acetone or glutaraldehyde to increase β-sheet crystallinity or cross-link the material to improve its water stability [130,132,135,140,142,143]. Antimicrobial agents that have been incorporated into SF-based fibers include a range antibiotics [131,133,138,139], antimicrobial peptides [132], antimicrobial nanoparticles [130,135] as well as natural substances such as essential oils and their components [127,131,137] and manuka honey [144]. Moreover, SF fibers have been functionalized with antibacterial nanoparticle coatings [140,142,143]. In a complex wound healing study that is worth mentioning, the authors have incorporated epidermal growth factor (EGF) into SF-PVA composite fibers, which were coated with ciprofloxacin hydrochloride to achieve enhanced wound healing. The fibers were tested in a rabbit wound model, and accelerated wound healing, enhanced re-epithelialization, and lead to highly vascularized granulation tissue and higher wound maturity in the case of fiber mats from non-mulberry SFs as compared to *Bombyx mori* SF-based mats [131]. In a second study, the authors have made similar observations when they incorporated the antimicrobial peptide LL-37 and EGF into the SF-PVA fibers [132].

### 4.5. Collagen and Gelatin

Collagen is the most abundant protein in mammals and the main component of the ECM in different organs and tissues including skin, bone, blood vessels, tendon and ligaments. Collagen fibrils are composed of cross-linked tropocollagen units that comprise three polypeptide chains that form a right-handed triple helix stabilized by interstrand hydrogen bonding and intrastrand n→π interactions. Cross-linking between adjacent tropocollagen units (length ≤ 300 µm) stabilizes the growing collagen fibrils (length ≤ 1 cm). Collagen plays a vital role in maintaining the biological and structural integrity of the ECM through its high tensile strength and mechanical resilience. Of the 29 collagen types of collagen, only a few fibril-forming, in particular collagen I, are used in the production of collagen-based biomaterials. Different inherent properties such as biodegradability, weak antigenicity, controllable mechanical properties, interaction with different cell types and formation of three-dimensional scaffolds, make collagen a relevant material for tissue-engineering and clinical applications [173,174]. During electrospinning care needs to be taken as denaturation of the collagen conformation may occur as a result of high voltage. Collagen is often co-electrospun with other synthetic polymers such as PCL to increase the stability of the fibers. In vivo, biodegradability of collagen fibers is achieved by endogenous collagenases, such as matrix metalloproteinases.

Gelatin is a soluble protein obtained by partial alkaline, acid or enzymatic hydrolysis of porcine, bovine or fish collagen. The manufacturing process has an impact on the properties of gelatin such as molecular weight and isoelectric point, with type A gelatin from acidic pre-treatment and type B gelatin from basic hydrolysis showing isoelectric points of 8–9 and 4–5, respectively. Gelatin dissolves in hot water and forms gels on cooling. The sol-gel transition occurs at temperatures between < 20 °C and 30 °C, and gelation involves a partial restoration of the triple helices of collagen in the gelatin polymeric chains. Gelatin is widely used in electrospinning due to its biodegradability, easy availability, low antigenicity, versatile usability and low cost. Due to the presence of the RGD motif, gelatin—just like collagen—shows the ability for cell attachment through integrin-mediated interactions. Due to its solubility, gelatin needs to be cross-linked to increase its stability and the stability of electrospun fibers in aqueous solutions [175].

There are various studies that have used collagen and gelatin as a starting material for electrospun wound dressings. Collagen has been electrospun alone [116], together with synthetic polymers PCL [115,118], PEO [118] and PLA/PLGA [25,112,113,114] as well as the natural polymer CS [117]. Gelatin has also been electrospun alone [120], in combination with synthetic polymers such as PVA [122] and poly([2-(methacryloyloxy)ethyl] trimethylammoniumchloride) (PMETAC) [121] and together with the natural polymer alginate dialdehyde [119] (Table 2). In some cases, electrospun collagen-, and in particular, gelatin-based fibers have to be cross-linked, for instance through exposure to ethanol [119] or glutaraldehyde [116,121,122] to improve the material’s water stability. Antimicrobial agents that have been incorporated into collagen- and gelatin-based fibers include a wide range of antibiotics [25,112,113,114,118,119,120], antibacterial nanoparticles [116,117] as well as plant extracts [122]. Interestingly, in one study wound dressings were prepared from mixtures of PCL and collagen in different ratios, and a virus (enterobacteria phage T4) was incorporated into the fibers. Systems composed of 30% PCL and 70% collagen with the virus found to be most promising as a hemostatic wound dressing with bacterial antibiotic function [115].

### 4.6. Elastin, Tropoelastin and Elastin-Like Recombinamers

Elastin, the core protein of elastic fibers, is an essential protein of the ECM of vertebrates, which imparts elasticity and resilience to organs and tissues such as blood vessels, lung, skin, ligaments and cartilage. It is composed of extensively cross-linked units of its precursor tropoelastin. Elastin shows unique properties such as a half-life of >70 years, extreme durability, reversible stretchability and resistance towards chemical and mechanical influences. The Young’s modulus of elastic fibers ranges between 300 kPa and 600 kPa, and they can be stretched up to 220% of their original length and undergo billions of cycles of extension and recoil without failing [176]. Elastin is able to mediate cell interactions such as dermal fibroblast attachment and spreading via interaction with integrins αVβ3 and αVβ5 [177], which is useful for wound healing purposes. Elastin peptides, which occur as a result of enzymatic elastin cleavage during aging, disease and physiological processes, display a number of bioactive effects when released into the blood stream, including stimulation of proliferation, protease expression, vasodilatation, apoptosis as well as chemotaxis of smooth muscle cells, endothelial cells and monocytes [178]. These may be used in a targeted way in the context of wound healing.

Elastin is challenging to work with as a starting material for the fabrication of biomaterials by electrospinning due to its resistance and insolubility. However, elastin from bovine and porcine tissues can be pre-treated by acidic or basic hydrolysis forming α- and κ-elastin, respectively, which improves elastin’s solubility. Drawbacks of such preparations are their heterogeneity in composition, and they may lose their cellular signaling ability, and require cross-linking by glutaraldehyde vapors to improve the water stability of the fiber mats. In recent years, recombinant human tropoelastin has been increasingly used for the fabrication of biomaterials for various tissue-engineering applications due to ethical concerns associated with the use of elastin from animals. Moreover, elastin-like peptides and artificial silk-elastin-like proteins (SELPs) are increasingly being used due to the possibility to control the amino acid sequences, chain lengths as well as chemical, mechanical and biological properties [179,180,181]. SELPs contain several repeats of silk- and elastin-like blocks based on conserved amino acid motifs of the two native proteins SF and elastin, and they combine the high tensile strength of SF with the elasticity and resiliency of elastin. While the SF units self-assemble into packed antiparallel β-sheet structures of great mechanical strength, the elastin-like building blocks display high flexibility [181]. Examples for electrospun elastin-based systems with potential applications in dermal tissue replacement and wound healing are given in Table 3.

To the best of our knowledge, no antimicrobial agents have so far been incorporated into such elastin-based fibers; however, focus was placed on stimulating the actual wound healing process by providing scaffolds that mimic the physicochemical properties of native skin ECM and support invasion, attachment and growth of cells and, hence, wound closure. It has indeed been shown in vitro in cell culture [182,183,184,185,186] and in animal models [182] that such scaffolds accelerate wound healing through increasing fibroblast invasion, attachment and proliferation. In particular, composite fibrous mats containing elastin/tropoelastin and collagen proved to be beneficial as the properties of both elastin (elasticity) and collagen (tensile strength) are combined. In such formulations, elastin decreases the stiffness of the scaffold, thus improving its mechanical properties [182,184,186].

## 5. Opportunities and Challenges of Using Proteins for Electrospun Drug Delivery Systems for Wound Healing

As mentioned previously, there are a number of benefits that proteins display when used as antimicrobial delivery systems. Most importantly, proteins show increased cytocompatibility and higher rate of biodegradation as compared to synthetic polymers [24,34]. Irrespective of their origin (plant or animal), proteins represent natural biomolecules that are degraded by physiological mechanisms [55]. For example, degradation of zein in vivo can be attributed to enzymatic and microbial activity as well as cell phagocytosis [188]. Another advantage of using proteins for antimicrobial drug delivery systems concerns their cell interaction abilities. Proteins such as soy protein [83], keratin, collagen, gelatin and elastin, for example, carry RGD or other cell-recognition motifs that facilitate recognition by cells, cell adhesion to fibers and cell migration across the wound bed (Figure 1). Proteins such as soy protein [88], lactoferrin [146] and lysozyme [98] further possess innate antimicrobial, anti-oxidant and anti-inflammatory activity that facilitate healing. The biological properties and further beneficial features of proteins of plant and animal origin that are used for electrospinning in the wound healing context are summarized in Table 4.

Using plant- and animal-derived proteins in electrospinning nanofibers not only comes with benefits, but also with a few challenges. To begin with, the diversity in extraction and purification methods has been shown to affect purity, composition and activity of the proteins. This in turn may influence the reproducibility during electrospinning, which is highly dependent on the homogeneity and surface charge of the material [5,36,37,38,57]. Unfortunately, there are currently no regulations in place to ensure homogeneity of structure and purity of protein-based materials [5]. Secondly, the use of organic solvents, cross-linking agents and a high voltage that are required during electrospinning may potentially inflict damage on the protein structure, which may result in loss of activity to a certain degree [22,23]. Even though there are some hydrophilic proteins such as casein, whey and SF (Table 2), using water as a solvent for electrospinning is a challenge in itself due to its surface tension that often leads to non-continuous process and artifacts in fibers. Moreover, aggregation and the low degree of protein unfolding in water further reduces protein electrospinnability [29]. And even if electrospinning is successful, the final product often requires cross-linking to ensure stability of fibers in water. For proteins with more amphiphilic nature such as zein, which can be dissolved in aqueous solvents, the final product loses its fibrous structure upon contact with water and becomes more elastic [54].

Regarding the incorporation of antimicrobial agents into protein-based fibers, a high diversity of antimicrobial agents can be integrated into fibers and elicit efficient antimicrobial response against Gram-negative and Gram-positive bacterial species (Table 1 and Table 2). Among plant-derived proteins, only three proteins (zein, soy and pea) have been reported to be electrospun with antimicrobial agents. Zein is the most popular protein to electrospin with a wide variety of antimicrobial agents, mostly including antibiotics and silver nanoparticles (Table 1). This may be attributed to its high availability as a manufacturing by-product and the associated low price [57] (Table 4). Among animal-derived proteins, collagen/gelatin as well as SF are widely used in a wound healing context both alone (which requires cross-linking) and in combination with synthetic polymers including PCL, PVA, PEO, PL(G)A and CS (Table 2). Antimicrobial agents that have been incorporated into such fibers include antibiotics, antimicrobial nanoparticles, antimicrobial peptides, antibacterial plant extracts and even an antibacterial virus [115] (Table 2). In the case of some fibrous mats made from SS or SF, instead of adding an antimicrobial agent, electrospinning with antibacterial polymers such as CS proved to be enough to confer the electrospun wound dressing with an antibacterial activity [123,126,128]. Overall, zein, gelatin and SF are the only proteins that can be electrospun without toxic organic solvents. However, due to their instability in water, all of them require either post-electrospinning cross-linking or the addition of synthetic polymers during electrospinning.

In summary, selecting proteins for electrospinning with antimicrobial agents depends not only on the ease of electrospinnability and properties required to facilitate wound healing, but also on the protein origin, availability, cost, manufacturing, purity, composition and allergenicity. It is worth noting that proteins with low nutritional value and those which occur as manufacturing by-products represent the most cost-effective and ecologically friendly option for producing nanofibers. Developing wound dressings from naturally-derived materials by upcycling manufacturing by-products and products with low nutritive value will help in establishing sustainable wound management approach with economically and environmentally friendly alternative to synthetic polymers.

## Figures and Tables

**Table 1:** Electrospun plant-based proteins with antimicrobial activity.

Protein	Co-Polymer	Electrospinning Type	Solvent	Antimicrobial Agent	Tested Bacterial Strain	Reference
Pea	PVA, CA	Uniaxial	Water	CA	*E. coli*, *L*. *monocytogenes*	[43]
Soy	PEO	Uniaxial	NaOH	None	*S. aureus*, *P*. *aeruginosa*	[44]
Zein	None	Co-axial	AA	ATPPB	*E*. *coli*, *S*. *aureus*	[45]
Zein	PU/CA	Uniaxial	DMF, MEK	Streptomycin	*E. coli*, *S. typhimurium*, *V*. *vulnificus*, *S*. *aureus*, *B*. *subtilis*	[26]
Zein	HA	Uniaxial	TFE, AA	Salicylic acid	*S. aureus*	[46]
Zein	PU	Uniaxial	DMF, THF	Ag NPs	*E. coli*, *S. aureus*	[27]
Zein	PCL, GA	Uniaxial	FA, AA	GA	*E. coli*, *S. aureus*	[47]
Zein	PCL, GA	Uniaxial, multilayer	FA, AA	GA, C. officinalis	*E. coli*, *S. aureus*	[48]
Zein	PCL	Uniaxial	TFE, DCM	Tetracycline hydrochloride	MRSA	[49]
Zein	None	Uniaxial	EtOH, water	Ag NPs	*E. coli*, *S. aureus*	[50]
Zein	None	Uniaxial	EtOH, water	Gentamicin	*E. coli*, *S. aureus*	[51]
Zein	None	Uniaxial	EtOH, water	Ag NPs	*E. coli*, *Bacillus*	[52]
Zein	None	Co-axial	EtOH, water	OEO	*E*. *coli*	[53]
Zein	PEO	Co-axial	EtOH, water	Tetracycline hydrochloride	*E*. *coli*, *S. aureus*	[54]
Zein	GT, PLA	Uniaxial	EtOH, water, CHL	Tetracycline hydrochloride	*S*. *aureus*, *P*. *aeruginosa*	[55]

Key: AA, acetic acid; ATPPB, allyltriphenylphosphonium bromide; CA, cinnamaldehyde; CHL, chloroform; DCM, dichloromethane; DMF, N,N-dimethylformamide; EtOH, ethanol; FA, formic acid; GA, gum arabic; GT, gum tragacanth; HA, hyaluronic acid; MEK, methyl ethyl ketone; MRSA, methicillin-resistant *S. aureus*; NaOH, sodium hydroxide; OEO, orange essential oil; PLA, polylactic acid; PU, polyurethane; TFE, 2,2,2-trifluoroethanol; THF, tetrahydrofuran.

**Table 2:** Electrospun animal-based proteins with antimicrobial activity.

Protein	Co-Polymer	Electrospinning Type	Solvent	Antimicrobial Agent	Tested Bacterial Strain	Reference
Casein	PEO	Uniaxial	Water	Ampicillin	*E. coli*, *S. aureus*	[99]
α-lactoglobulin	PEO	Uniaxial	Water	Ampicillin	*E. coli*, *P. aeruginosa*, *B. thailandensis*	[107]
Lactoferrin	Gelatin	Uniaxial	FA, DMF	None	*E. coli*, *S. aureus*	[108]
Lysozyme	CS, PVA	Uniaxial	AA, water	CS	*S. aureus*, *B. subtilis*, *S. flexnery*, *P. aeruginosa*	[109]
Keratin	PVA, PEO	Uniaxial	NaOH	Ag NPs	*E. coli*, *S. aureus*	[110]
Keratin	CS, PHBA, gelatin	Uniaxial, multilayer	HFIP	Mupirocin	*E. coli*, *S. aureus*	[111]
Collagen	PLGA	Uniaxial, multilayer	HFIP	Vancomycin hydrochloride, gentamicin sulfate	*E. coli*, *S. aureus*	[112,113,114]
Collagen	PCL	Uniaxial	HFIP	Enterobacteria phage T4	*E. coli*	[115]
Collagen	PLA	Uniaxial	HFIP	Levofloxacin	*E. coli*, *S. aureus*	[25]
Collagen	-	Uniaxial	HFIP	Ag NPs	*S. aureus*, *P. aeruginosa*	[116]
Collagen	CS	Uniaxial	0.5 M AA	ZnO	*S. aureus*, *E. coli*	[117]
Collagen	PCL (core), PEO (shell)	Co-axial	HFIP, glacial AA	Doxycycline	*n.a.*	[118]
Gelatin	Alginate-dialde-hyde	Uniaxial	AA(40% w/w)	Ciprofloxacin, gentamicin	*P. aeruginosa*, *S. epidermidis*	[119]
Gelatin	-	Uniaxial	TFE	Vancomycin, caspofungin	MRSA, *C. albicans*	[120]
Gelatin	PMETAC	Uniaxial	FA, AA	PMETAC	*S. aureus*, *E. coli*, MRSA, *A. baumannii*	[121]
Gelatin	PVA	Uniaxial	FA	*Centella asiatica* extract	*S. aureus*, *E. coli*, *P. aeruginosa*	[122]
Silk sericin	CS	Uniaxial	TFA	CS	*E. coli*, *B. subtilis*	[123]
Silk sericin	PLLA	Uniaxial, multilayer	TFA	Nitrafurazone	*E. coli*, *B. subtilis*	[124]
Silk sericin	CS, PVA	Uniaxial	Water	Cephalexin hydrate	*E. coli*, *B. subtilis*	[125]
Silk fibroin	PCL	Uniaxial, multilayer	HFIP	CS	*S. aureus*, *E. coli*	[126]
Silk fibroin	-	Uniaxial	FA	Oleuropein	*S. epidermidis*, *E. coli*	[127]
Silk fibroin	CS	Uniaxial	HFIP, TFE	CS	*S. aureus*, *E. coli*	[128]
Silk fibroin, sulfated fibroin	PEI	Uniaxial	FA	PEI	*S. aureus*, *P. aeruginosa*	[129]
Silk fibroin	-	Uniaxial	FA	Ag NPs	*S. aureus*, *P. aeruginosa*	[130]
Silk fibroin	PVA	Uniaxial	Water	EGF, ciprofloxacin hydrochloride	*S. aureus*, *S. epidermidis*, *E. coli*, *P. aeruginosa*	[131]
Silk fibroin	PVA	Uniaxial	Water	LL-37 antimicrobial peptide, EGF	*S. epidermidis*, *P. aeruginosa*	[132]
Silk fibroin	Gelatin	Uniaxial	FA	Thyme essential oil, doxycycline monohydrate	*S. aureus*, *K. pneumoniae*	[133]
Melamine-modified silk fibroin	PCL	Uniaxial	HFIP	Melamine-modified silk fibroin	*S. aureus*, *E. coli*	[134]
Silk fibroin	PEO	Uniaxial	FA	TiO_2_ NPs	*E. coli*	[135]
Silk fibroin	P(LLA-CL)	Uniaxial	HFIP	Curcumin	*S. aureus*	[136]
Silk fibroin	PCL, HA, PEO	Uniaxial, multilayer	FA, TFE, water	Thymol	*S. aureus*, *P. aeruginosa*	[137]
Silk fibroin	CS, halloysite nanotubes, PEO	Uniaxial	FA, AA, water	Chlorhexidine digluconate	*S. aureus*, *E. coli*	[138]
Silk fibroin	Gelatin	Uniaxial	FA	Ceftazidime	*P. aeruginosa*	[139]
Silk fibroin		Uniaxial	FA	Selenium NP coating	*S. aureus*	[140]
Silk fibroin	Carboxy-methyl CS coating	Uniaxial	HFIP, AA	Carboxymethyl CS coating	*S. aureus*, *E. coli*	[141]
Silk fibroin		Uniaxial	HFIP, FA	Ag NP coating	*S. aureus*, *P. aeruginosa*	[142]
Silk fibroin		Uniaxial	FA, water	Graphene oxide coating	*S. aureus*, *E. coli*	[143]
Silk fibroin	PEO	Uniaxial	Water	Manuka honey	MRSA, *P. aeruginosa*, *E. coli*, *S. aureus*	[144]
Silk fibroin	PEO	Uniaxial	Water	Cu_2_O NPs	*S. aureus*, *E. coli*	[145]

Key: AA, acetic acid; Ag NPs, silver nanoparticles; CS, chitosan; DMF, N,N-dimethylformamide; EGF, epidermal growth factor; FA, formic acid; HA, hyaluronic acid; HFIP, 1,1,1,3,3,3-hexafluoro-2-propanol; MRSA, methicillin-resistant *S. aureus*; NP, nanoparticle; PBS, phosphate buffered saline; PCL, polycaprolactone; PEI, polyethylenimine; PMETAC, poly([2-(methacryloyloxy)ethyl] trimethylammoniumchloride); PEO, polyethylene oxide; PHBA, poly(3-hydroxybutyric acid); PHBV, poly(3-hydroxybutyrate-co-3-hydroxyvalerate); PLLA, poly(L-lactic acid); P(LLA-CL), poly(L-lactic acid-co-e-caprolactone); TFA, trifluoroacetic acid; TFE, 2,2,2-trifluoroethanol.

**Table 3:** Electrospun elastin-based fiber mats for wound healing applications.

Protein	Co-Polymer	Electrospinning Type	Solvent	Potential Applications	Reference
Elastin	Collagen, PCL	Uniaxial	HFIP	Skin grafting, dermal substitute for burn wounds	[182]
ELR	-	Uniaxial	TFE	Wound dressings, skin tissue engineering	[183]
Elastin	Gelatin, CA	Uniaxial	AA	Skin injuries caused by trauma and diseases	[184]
SELPs	-	Uniaxial	Water, FA	Wound dressings, skin regeneration	[181]
Tropoelastin	-	Uniaxial	HFIP	Dermal tissue engineering	[185]
Tropoelastin	Collagen	Uniaxial	HFIP	Dermal tissue engineering	[186]
SELPs	Silk fibroin	Co-axial	Water	Biomedical applications, drug delivery	[187]

Key: AA, acetic acid; CA, cellulose acetate; ELR, elastin-like recombinamer; FA, formic acid; HFIP, 1,1,1,3,3,3-hexafluoro-2-propanol; PCL, polycaprolactone; SELP, silk-elastin-like protein; TFE, 2,2,2-trifluoroethanol.

**Table 4:** Biological properties and other relevant features of plant- and animal-derived proteins.

Protein	Water Soluble	Nutritional Value	Indu- strial By- Product	Aller- genic	Anti- microbial	Anti-oxi-dant	Anti-Inflamma-tory	Cell-Recog-nizing Motifs	Self-Assem-bly
Zein			✓			✓			✓
Soy		✓	✓	✓	✓	✓	✓	✓	
Pea	✓	✓	✓	✓					
Casein	✓	✓	✓	✓					✓
α-lacto-globulin	✓	✓							✓
β-lacto-globulin	✓	✓							✓
Lacto-ferrin	✓	✓			✓	✓	✓		
Lysozyme	✓	✓			✓	✓	✓		
Keratin			✓					✓	✓
Collagen		✓		(✓)				✓	✓
Gelatin	✓	✓		(✓)				✓	✓
Elastin		✓		(✓)				✓	✓
Silk sericin				(✓)	✓	✓			✓
Silk fibroin	✓			(✓)				(✓)	✓

Note: ✓, present property; (✓) weak manifestation of property; blank cell, not reported.
